# Renewable synthesis of γ-butyrolactone from biomass-derived 2-furanone using palladium supported on humin-derived activated carbon (Pd/HAC) as a heterogeneous catalyst

**DOI:** 10.1039/d3ra01377d

**Published:** 2023-05-17

**Authors:** Nivedha Vinod, Saikat Dutta

**Affiliations:** a Department of Chemistry, National Institute of Technology Karnataka (NITK) Surathkal Mangalore-575025 India sdutta@nitk.edu.in

## Abstract

This work reports a high-yielding synthesis of γ-butyrolactone (GBL), a promising biofuel, renewable solvent, and sustainable chemical feedstock, by the catalytic hydrogenation of 2-furanone. 2-Furanone can be synthesized renewably by the catalytic oxidation of xylose-derived furfural (FUR). Humin, produced during the preparation of FUR from xylose, was carbonized to form humin-derived activated carbon (HAC). Palladium supported on humin-derived activated carbon (Pd/HAC) was used as an efficient and recyclable catalyst for hydrogenating 2-furanone into GBL. The process was optimized in various reaction parameters, such as temperature, catalyst loading, hydrogen pressure, and solvent. Under optimized conditions (RT, 0.5 MPa H_2_, THF, 3 h), the 4% Pd/HAC (5 wt% loading) catalyst afforded GBL in an 89% isolated yield. Under identical conditions, an 85% isolated yield of γ-valerolactone (GVL) was obtained starting from biomass-derived angelica lactone. Moreover, the Pd/HAC catalyst was conveniently recovered from the reaction mixture and successfully recycled for five consecutive cycles with only a marginal decrease in the yield of GBL.

## Abstract 2

A palladium catalyst supported on humin-derived activated carbon produced γ-butyrolactone and γ-valerolactone from 2-furanone and angelica lactone, respectively, in excellent yields.

## Graphical abstract

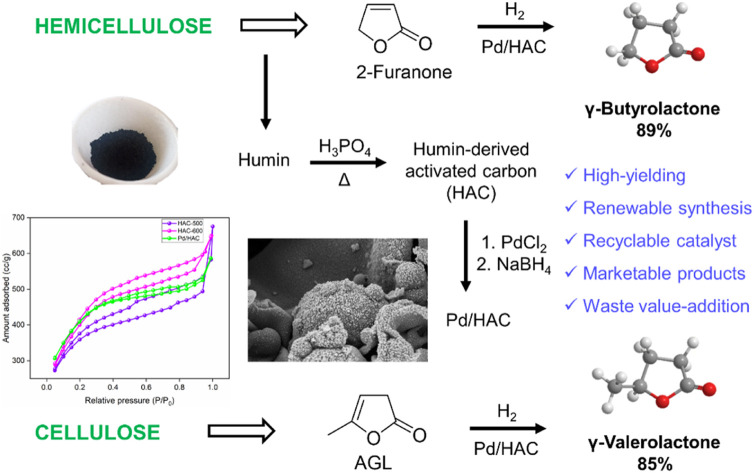

## 1 Introduction

There are significant societal, economic, and environmental incentives for producing liquid transportation fuels and organic chemicals from biomass as a source of biogenic organic carbon to alleviate the overdependence on anthropogenic carbon.^1,2^ In a biorefinery setting, various biomass components are transformed into organic fuels and chemicals of desired molecular structures and properties.^3^ Lignocellulosic biomass has received particular attention since it is geographically diverse, abundant, inexpensive, and often found in various waste streams.^4,5^ Catalysis is a cornerstone of green chemistry and an integral part of a modern biorefinery for the selective conversion of biomass components into bulk and specialty organic chemicals under commercially feasible and environmentally acceptable conditions.^6^ Acid hydrolysis of lignocellulosic biomass is an established catalytic strategy that not only allows the biopolymers to separate from each other but also deconstructs the biopolymers into small organic molecules.^7^ For example, the hemicellulose fraction leads to furfural (FUR), whereas the cellulose fraction forms levulinic acid (LA) under acid catalysis at elevated temperatures.^8,9^ FUR and LA have been established as carbohydrate-derived chemical platforms for producing bulk- and specialty chemicals that are otherwise sourced from petroleum.^10–12^ FUR can be catalytically oxidized to 2-furanone using innocuous oxidants, such as gaseous O_2_ and aqueous H_2_O_2_.^13,14^ Catalytic hydrogenation of the olefinic group in 2-furanone leads to γ-butyrolactone (GBL).^15^ GBL has applications as an industrial solvent, potential biofuel, and chemical feedstock in several industries, such as polymers, cosmetics, and pharmaceuticals.^16,17^ GBL is commercially produced by dehydrogenating 1,4-butanediol over a copper catalyst or by partially reducing maleic anhydride over a nickel catalyst.^18^ Even though the processes mentioned above can utilize both petroleum and biomass resources, the synthetic routes involve multiple synthetic steps and demanding reaction conditions.

**Scheme 1:**
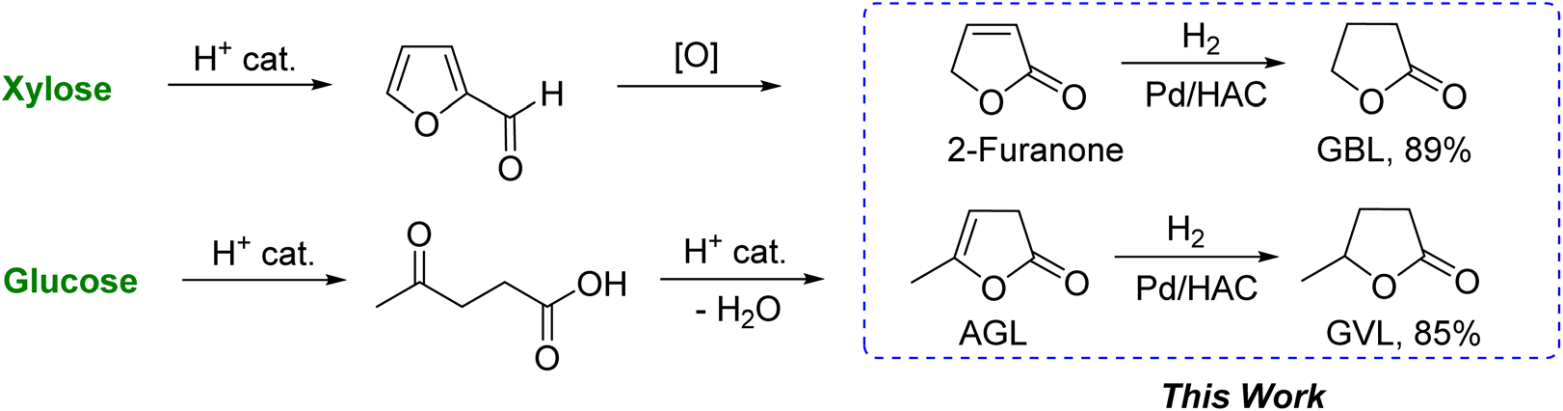
Catalytic synthesis of γ-butyrolactone and γ-valerolactone from carbohydrates.

The intramolecular dehydrative lactonization of LA leads to angelica lactones (AGLs), a combination of three positional isomers.^19,20^ Catalytic hydrogenation of the olefin group in AGLs leads to γ-valerolactone (GVL).^21^ GVL has received interest as a diesel additive, renewable solvent, and chemical feedstock.^22^ Among the various noble metals and non-noble metal catalysts examined, palladium is one of the most active metals for the catalytic hydrogenation of 2-furanone to GBL, and AGLs to GVL, respectively.^23,24^ Activated carbon (AC), possessing high surface area and hierarchical pore structures, is a commonly employed supporting material for metal-based heterogeneous catalysts.^25^ In this regard, humin is a complex furanic resin that forms inadvertently during the production of FUR and LA from sugars and polymeric carbohydrates.^26^ Humin, in principle, could act as an inexpensive precursor for deriving AC for catalyst support. At present, humin is typically combusted as a solid fuel to generate process heat for other biorefinery processes or to produce synthesis gas.^27^ Alternative value-addition pathways for humin, such as reinforcing materials, adsorbent, and catalyst support, have attracted significant interest recently.^28–30^ In this work, water-insoluble humin produced during the dehydration of xylose into FUR was used as the starting material to form the carbon-based catalyst support. Humin was carbonized and activated by treating with orthophosphoric acid (H_3_PO_4_) at 600 °C. Palladium metal was supported on humin-derived activated carbon (HAC) by the wet impregnation method. The Pd/HAC catalyst was employed as a heterogeneous catalyst for hydrogenating 2-furanone and α-AGL into GBL and GVL, respectively (Scheme 1).

## 2 Experimental section

### 2.1 Materials

Xylose (99%) and benzyltributylammonium chloride (BTBAC, 99%) were procured from Spectrochem. PdCl_2_ was purchased from Sigma. NaBH_4_, 1,2-dichloroethane (DCE) (98%), HCl (aq., 35–38%), and tetrahydrofuran (THF) (99.5%) were obtained from Molychem. Sodium sulfate (anhydrous, 99%), NaOH, and deionized (DI) water were purchased from Loba Chemie Pvt. Ltd. Methanol (99.5%) and chloroform (98%) were purchased from Finar. AGL (>95% α-isomer) was purchased from Alfa Aesar and purified by passing through a plug of silica gel (60–120 mesh) using chloroform as the eluent. Chloroform was then evaporated in a rotary evaporator under reduced pressure to obtain pure α-AGL as a faint-yellow oil. 2-Furanone was synthesized according to the literature procedure.^14^

### 2.2 Characterization methods

Fourier-transform infrared spectroscopy (FTIR) spectra of the HAC support and Pd/HAC catalyst were recorded on a Bruker Alpha 400 FTIR spectrometer using the KBr pellet technique. Powder X-ray diffraction (PXRD) patterns were recorded on Rigaku MiniFlex 600 (Japan) X-ray powder diffractometer using Cu Kα radiation as the source. The surface morphology of the samples was investigated using field emission scanning electron microscopy (FESEM), Gemini 300, Carl Zeiss, operating at an accelerating voltage of 15 kV, and the high-resolution transmission electron microscopy (HR-TEM) images were recorded on the FEI Tecnai G2 F30 S-Twin instrument. Brunauer–Emmett–Teller (BET) surface area of the samples was measured using Autosorb IQ-XR-XR, Anton Paar, employing N_2_ adsorption at 77.35 K. The pore size distribution of the samples was determined from nitrogen desorption isotherms using the Barrett–Joyner–Halenda (BJH) method. Thermogravimetric analysis (TGA) was recorded on a TGA 4000 (PerkinElmer) instrument under a nitrogen atmosphere at a temperature ramp of 10 °C min^−1^.

### 2.3 Preparation of humin

Humin was obtained as the side product during the dehydration of xylose into FUR in an aqueous–organic biphasic batch reaction setup.^31^ A round-bottomed glass pressure reactor fitted with a Teflon screw-top was charged with xylose (2.00 g), aq. HCl (20 mL, 20.2%), and a magnetic bead. To the solution, DCE (20 mL) and BTBAC (10 wt% of xylose) were added. The reactor was then sealed, placed in a preheated (100 °C) oil bath, and stirred magnetically at 400 rpm for 3 h. The biphasic reaction mixture slowly turned from colorless to yellow to dark brown. After 3 h, the reactor was lifted from the oil bath and cooled in air. The reactor was opened carefully, and the reaction mixture was filtered through filter paper under a vacuum. The black solid residue collected on the filter paper was washed with DCE (25 mL) to remove soluble organic residues, followed by excess deionized water. The humin was dried in a hot-air oven for 24 h at 80 °C.

### 2.4 Preparation of HAC

Orthophosphoric acid was used for the chemical activation of humin. In a typical process, 2 g of humin was mixed intimately with 85% H_3_PO_4_ (1 : 3, w/w) in a porcelain crucible and kept for 2 h. The mixture was carbonized at 500 and 600 °C in a muffle furnace for 2 h under a flowing N_2_ atmosphere. When the mixture was carbonized at 700 °C, it combusted partially, even under nitrogen flow. After carbonized mass (500 and 600 °C) was cooled to RT, suspended in deionized water (100 mL), and ultrasonicated for 30 minutes. The suspension was then filtered under a vacuum and repeatedly washed with deionized water until the pH of the filtrate increased to 7. The prepared samples were dried at 60 °C for 12 h in a hot-air oven and further sonicated for 30 minutes in methanol for uniform dispersion. After removing methanol, the carbon was dried at 110 °C for 6 h, powdered in a mortar, and labeled as HAC-*X*, *X* corresponds to carbonization temperature.

### 2.5 Preparation of Pd/HAC catalyst

The catalyst was prepared by the wet impregnation method.^32^ In this method, HAC-600 (0.500 g) was dispersed by ultrasonication in HPLC-grade water (20 mL) for 15 min. Palladium(ii) chloride (0.042 g) was dissolved in a minimum quantity of HCl (5%, aq.) and added dropwise into suspended humin at RT under vigorous stirring. After complete addition, the stirring was continued for an additional 2 h. An aqueous solution of NaOH (10 M) was then added dropwise while stirring to adjust the pH in the range of 8–9. The mixture was stirred for 30 min, and NaBH_4_ (0.500 g) was added in portions over 30 min. The solution was stirred again for 1 h, filtered, and washed with DI water until the pH level dropped to 7. The catalyst was dried in a hot-air oven for 5 h at 110 °C before using it in the hydrogenation reactions.

### 2.6 Catalytic hydrogenation of renewable lactones

The Hastelloy-made high-pressure reactor was charged with 2-furanone (0.502 g, 5.9 mmol), catalyst (0.025 g, 5 wt%), and THF (55 mL). The reactor was closed, purged with H_2_ three times, and pressurized to 0.5 MPa of H_2_ pressure. The reaction was carried out at RT for 3 h. After the reaction, the reaction mixture was centrifuged to separate the suspended Pd/HAC catalyst. The THF solvent was evaporated under reduced pressure to get crude GBL as a colorless liquid. The crude product was then passed through a small plug of silica gel (60–120 mesh) to get pure GBL (0.469 g, 89%) as a colorless liquid. The identity and purity of synthesized GBL were confirmed by spectroscopic techniques. Spectroscopic data: ^1^H-NMR (CDCl_3_, 300 MHz) *δ* (ppm): 4.35 (t, 2H), 2.49 (t, 2H), 2.28 (m, 2H); ^13^C-NMR (CDCl_3_, 75 MHz) *δ* (ppm): 178.3, 68.7, 27.81, 22.1; FTIR (ATR, cm^−1^): 2957, 2923, 1771, 1035.

The same synthetic procedure was employed for the hydrogenation of α-AGL to GVL, except that the catalyst loading was increased to 8 wt% (compared to starting α-AGL). An 85% isolated yield of spectroscopically GVL was obtained as a clear liquid. Spectroscopic data: ^1^H-NMR (CDCl_3_, 300 MHz) *δ* (ppm): 4.58 (m, 1H), 2.47 (m, 2H), 2.31 (m, 2H), 1.75 (d, 3H); ^13^C-NMR (CDCl_3_, 75 MHz): *δ* (ppm): 173.3, 77.2, 29.6, 29, 20.9; FTIR (ATR, cm^−1^): 2982, 2929, 1770, 1095.

## 3 Results and discussion

### 3.1 Physicochemical characterization

Iodine number (IN) is routinely measured to determine the adsorption capacity of AC. The IN of the synthesized HACs was determined according to ASTM standards (Table 1).^33^ The IN of HAC-500 and 600 were calculated to be 727 mg g^−1^ and 853 mg g^−1^, respectively. A high IN typically indicates a high surface area of AC involving large micro-and mesoporous structures.

**Table 1:** Pore structure parameters of HAC-500, HAC-600, and Pd/HAC

Sample	IN (mg g^−1^)	*S*_BET_ (m^2^ g^−1^)	*V*_total_ (cm^3^ g^−1^)	*D*_avg_ (nm)
HAC-500	727	1203	0.32	3.4
HAC-600	853	1425	0.33	2.8
Pd/HAC	—	1401	0.24	2.6

**Fig. 1:**
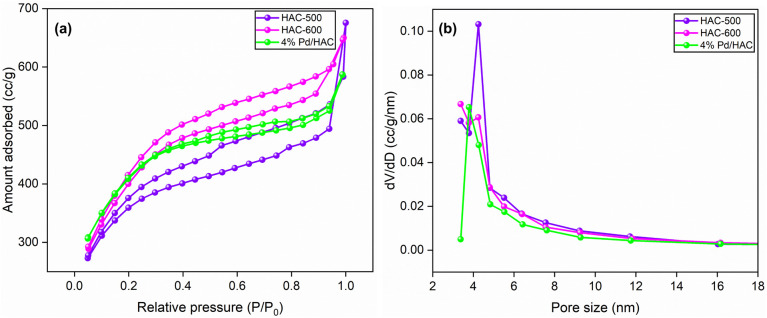
(a) N_2_ adsorption isotherms, (b) BJH pore size distribution curves of HAC-500, HAC-600, and Pd/HAC.

The textural characterization of the synthesized HACs was studied using nitrogen adsorption–desorption analysis. From the BJH pore size distribution curves, the pore size ranged between 2 nm and 40 nm. A distinct peak at 4.25 nm confirms that the materials are mesoporous with uniform pore size distribution (Fig. 1). HAC-600 exhibited type IV adsorption isotherm and was used for the catalyst preparation because of its higher IN and BET surface area values (Table 1). The surface area of the catalyst decreased marginally owing to the impregnation of Pd NPs on the HAC support.

**Fig. 2:**
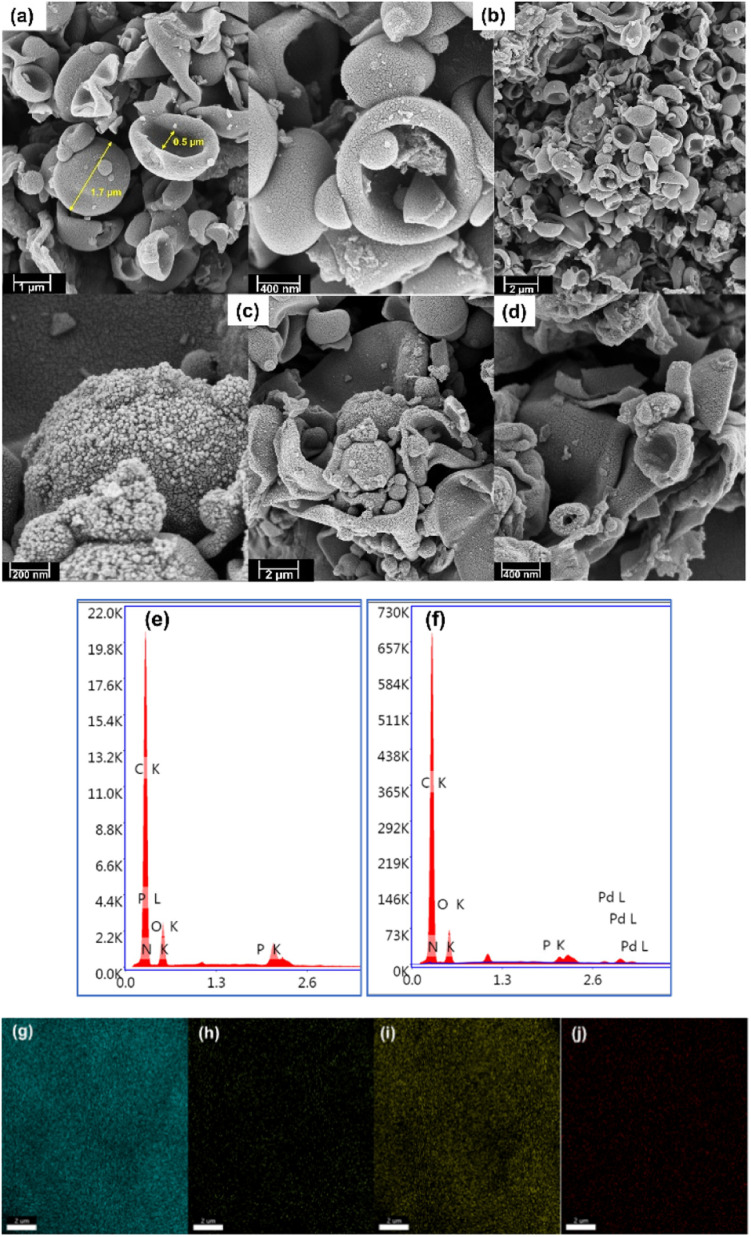
The FE-SEM images of (a) HAC-500, (b) HAC-600, (c) Pd/HAC (fresh), and (d) Pd/HAC (recovered after the 4th cycle). The EDX pattern of (e) HAC-600, (f) Pd/HAC, and elemental mapping of Pd/HAC for (g) C, (h) N, (i) O, and (j) Pd.

**Fig. 3:**
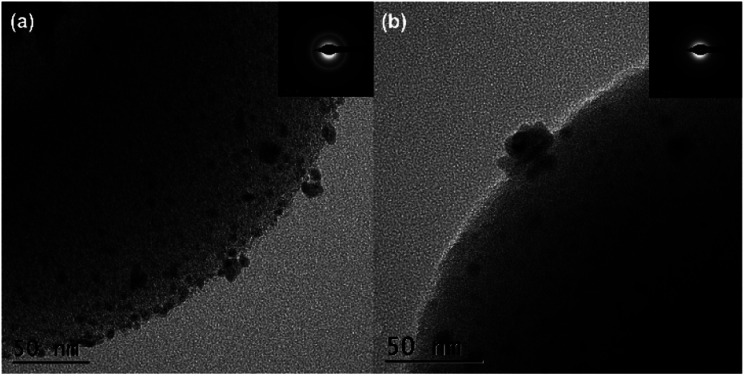
The HR-TEM images of (a) fresh Pd/HAC, (b) recycled Pd/HAC (after the 5th catalytic cycle), and their corresponding SAED pattern (inset).

The surface morphology of the materials was determined using field-emission scanning electron microscopy (FE-SEM). Fig. 2 shows the presence of cup-shaped structures in HAC. The distribution of Pd NPs over the HAC-600 support can be observed on the FE-SEM images, and HR-TEM also validated the data. The HR-TEM image of the recycled Pd/HAC catalyst (5th cycle) suggests agglomeration of Pd NPs and leaching from the HAC support (Fig. 3). The selected area electron diffraction (SAED) pattern shows the amorphous nature of the catalyst. The presence of constituent elements in the Pd/HAC catalyst was confirmed by energy-dispersive X-ray analysis (EDX) (e–j, Fig. 2). The elemental mapping of the catalyst further confirmed 4% loading of palladium and the uniform distribution of Pd NPs on the HAC support. The target of metal-on-carrier catalysts, such as the Pd/HAC catalyst reported in this work, is to ensure maximum metal dispersion on the supporting material. The extent of metal dispersion depends on multiple factors, including the metal loading, duration and temperature of reduction of the metal salt precursor, and texture of the carrier material. The uniformity of metal dispersion and free-metal surface area is a guideline to check the quality and reproducibility of different catalysts. The catalytic behavior (*e.g.*, activity, selectivity) of the metallic part of the catalyst relies on the texture, the structure, and the surface composition.^34^ For palladium catalysts, the extent of hydrogen adsorption depends on the metal dispersion.

**Fig. 4:**
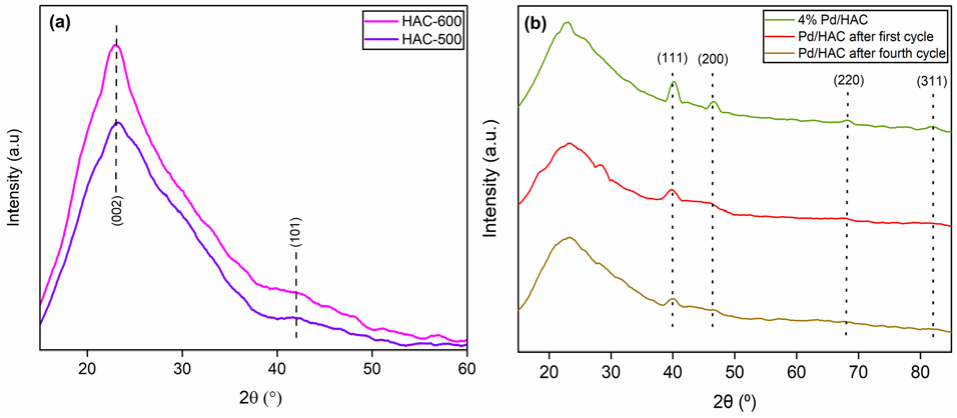
The PXRD patterns of (a) HAC-500 and HAC-600, (b) Pd/HAC (fresh), recycled after the first catalytic cycle, and recycled after the fourth catalytic cycle.

**Fig. 5:**
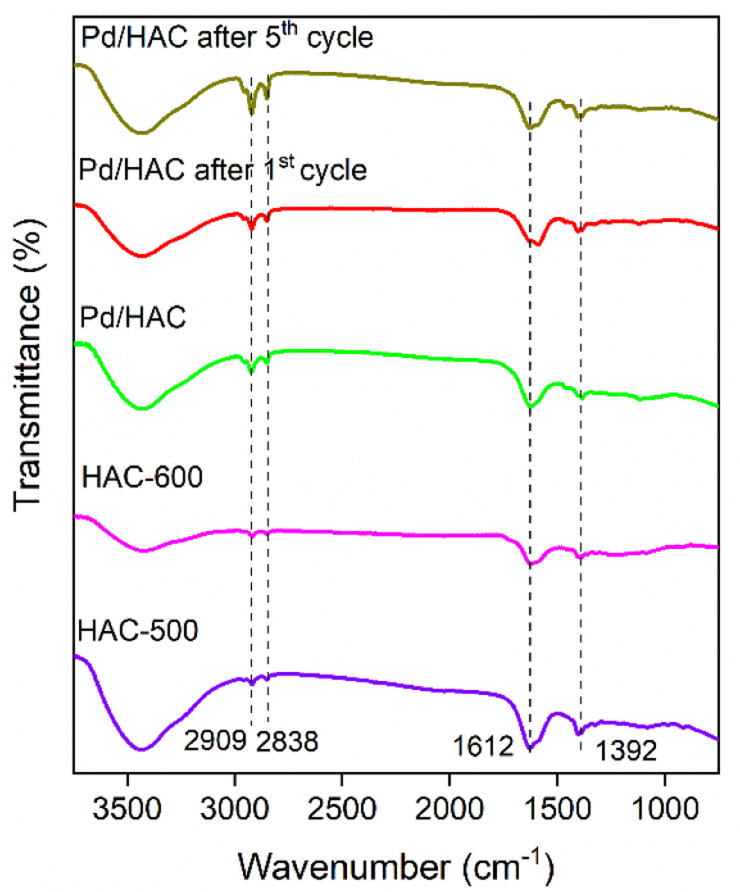
The FTIR spectra of HACs and the Pd/HAC catalysts (fresh and recycled).

**Fig. 6:**
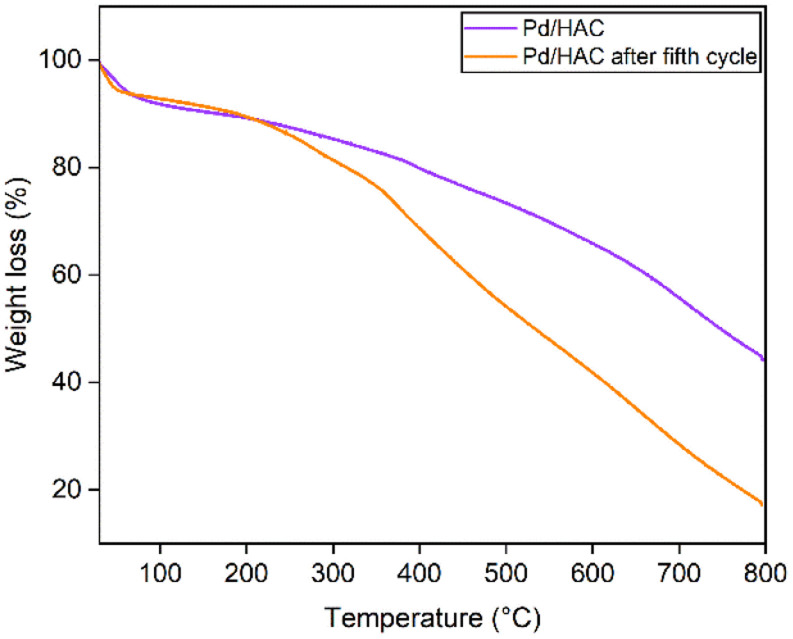
The TGA curves of fresh and recycled Pd/HAC catalyst.

The powder X-ray diffraction (PXRD) patterns of the HACs and catalyst are shown in Fig. 4. The scan was recorded in the 2*θ* range between 10–60°. A broad diffraction peak at 2*θ* = 23° confirms the amorphous nature of AC. It is evident from the literature that peaks located at 2*θ* = 23° and 43° for HAC-500 and 600 correspond to (002) and (101) planes of carbon. The PXRD pattern of the catalyst exhibit diffraction peaks at 40.1°, 46.5°, 67.8°, and 81.8°, corresponding to (111), (200), (220), and (311) planes of Pd, respectively (JCPDS card no. 46-1043). The peak at 40.1° suggests the formation of Pd nanoparticles. The FTIR spectra of the HACs and the catalyst are shown in Fig. 5. The bands 2909 and 2838 cm^−1^ correspond to C–H asymmetric and symmetric stretching, whereas bands assigned 1612 and 1392 cm^−1^ correspond to C=C stretch and C–H bending vibrations. Fig. 6 shows the thermal decomposition profiles of fresh and recycled Pd/HAC catalysts. A marginal weight loss till 90 °C corresponds to the loss in surface moisture content, and gradual decomposition of the catalyst is observed at higher temperatures. In the recycled Pd/HAC (5th cycle) catalyst, a faster weight loss was possibly due to the evaporation of volatiles chemisorbed on the catalyst surface.

### 3.2 Catalyst evaluation

**Fig. 7:**
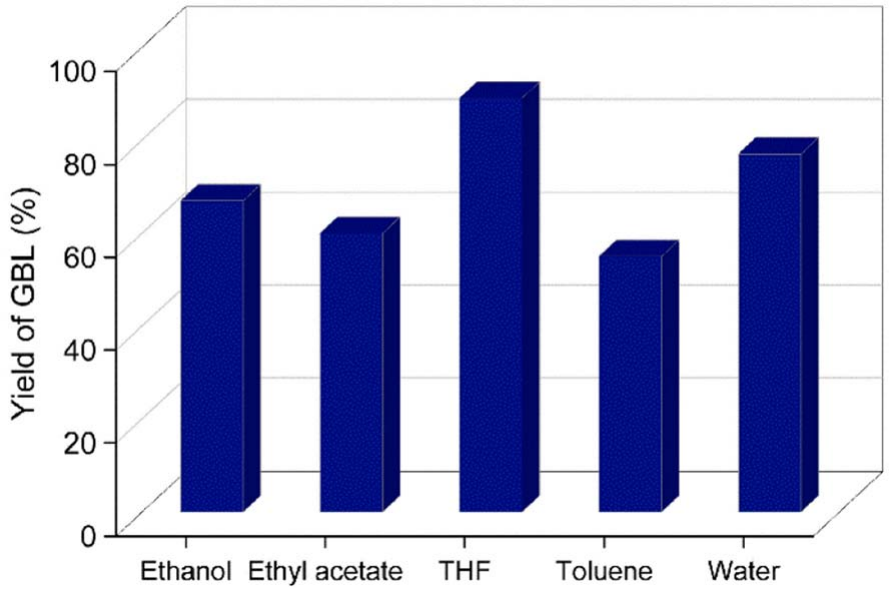
Effect of different solvents on the yield of GBL. Reaction conditions: 2-furanone (0.5 g), Pd/HAC catalyst (5 wt%, 0.025 g), solvent (55 mL), RT, 0.5 MPa H_2_, 3 h.

The prepared catalyst was evaluated for the hydrogenation of 2-furanone GBL in a Hastelloy-made high-pressure reactor. A complete conversion of 2-furanone was obtained when the reaction was carried out at RT for 3 h under 0.5 MPa H_2_ pressure. No conversion of 2-furanone was observed even after several hours when the reduction reaction was attempted using a hydrogen balloon. The yield of GBL remained the same when the reaction was carried out at 40 °C, keeping all other reaction parameters constant. The hydrogenation reaction was studied in various organic solvents. The solubility of hydrogen gas in the organic solvent is known to influence the outcome of the hydrogenation reaction. Ethereal solvents like THF are known to solubilize gaseous hydrogen better than aliphatic and aromatic hydrocarbon solvents.^35^ Different solvents were screened for the catalytic hydrogenation reaction, and a maximum GBL yield (*ca.* 89%) was obtained in the presence of THF (Fig. 7). The high yield of GBL in THF may be explained by the higher solubility of gaseous hydrogen in the solvent. Water was also examined as a green solvent for the catalytic hydrogenation reaction, and GBL was obtained in a 77% isolated yield. However, the high solubility of GBL and GVL in water somewhat defeats the purpose of using water as a sustainable reaction medium since an organic solvent is warranted to extract the product. When the reaction was carried out using commercially available 5% Pd/AC, GBL was obtained in 77% yield.

The catalyst was further evaluated for the hydrogenation of α-AGL to GVL. Interestingly, the conversion of α-AGL was incomplete under optimized reaction conditions, which may be attributed to the lesser reactivity of the olefinic group in α-AGL compared to 2-furanone towards catalytic hydrogenation. A complete conversion of α-AGL was obtained by doubling the catalyst loading (10 wt%). Further optimizations on the catalyst loading showed that an 8 wt% catalyst loading was enough to ensure quantitative conversion of α-AGL and a high isolated yield of GVL. The synthesized GBL and GVL were characterized using FTIR, ^1^H NMR, and ^13^C NMR spectroscopic techniques.

### 3.3 Recyclability of the catalyst

**Fig. 8:**
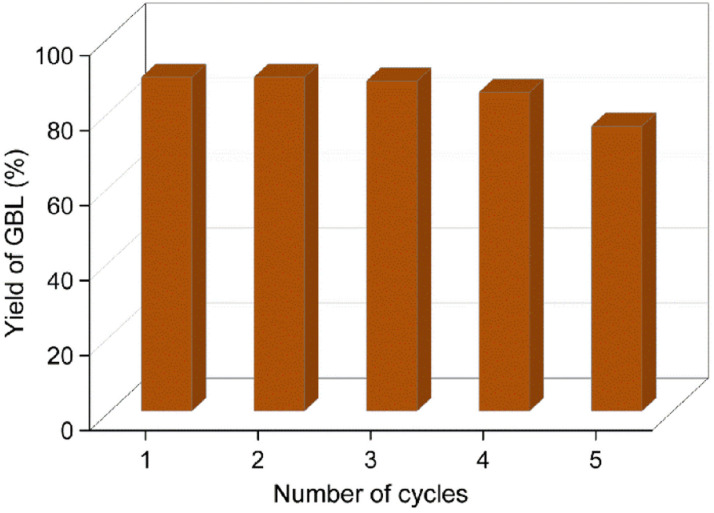
Catalytic recyclability test for the conversion of 2-furanone to GBL. Reaction conditions: 2-furanone (0.5 g), Pd/HAC catalyst (5 wt%, 0.025 g), THF (55 mL), RT, 0.5 MPa H_2_, 3 h.

After the reaction, the catalyst was centrifuged and reused for the next catalytic cycle after washing it with THF and dried in a hot-air oven at 80 °C for 12 h. The spent catalyst was characterized using FE-SEM, EDX, HR-TEM, TGA and PXRD techniques. The stability of the catalyst was excellent, with only a marginal drop in the catalytic activity after the fifth cycle. It is evident from the EDX data that the loss in catalyst activity was due to the leaching of the palladium metal particles (the amount of Pd decreased from 4% to 1.5%) and chemisorption of organic contaminants on the HAC support (Fig. 8). The authors envision that a glass-lined pressure reactor could slow down the leaching of Pd NPs from the HAC support and decrease the physical loss of the catalyst.

## 4 Conclusion

Palladium metal supported on humin-derived activated carbon showed excellent catalytic activity for the catalytic hydrogenation of furfural-derived 2-furanone in a batch reactor, providing an 89% isolated yield of GBL. The Pd/HAC catalyst also successfully hydrogenated angelica lactones into GVL, affording an 85% isolated yield. The starting materials, *i.e.*, α-AGL and 2-furanone, are known to form many reduced products in the presence of Pd-based catalysts under appropriate reaction conditions. However, in his work, the hydrogenation reaction was performed under mild conditions (*i.e.*, RT-40 °C, 0.5 MPa H_2_) so that only the olefin group in 2-furanone and α-AGL get reduced. The activation energy for the other competing mechanistic pathways is not reached under these conditions. The catalyst showed excellent recyclability and retained its activity till the third cycle. The yield of GBL decreased marginally to 76% after the fifth consecutive cycle. Deactivation of the catalyst is due to the partial leaching of Pd NPs from the HAC support and the chemisorption of organic contaminants on the catalyst surface. Future research will employ this catalyst for synthesizing other biorenewable fuels and chemicals by catalytic hydrogenation.

## Abbreviations

α-AGL: α-Angelica lactone
BTBAC: Benzyltributylammonium chloride
GBL: γ-Butyrolactone
DCE: 1,2-Dichloroethane
EDX: Energy-dispersive X-ray analysis
FE-SEM: Field-emission scanning electron microscopy
HR-TEM: High-resolution transmission electron microscopy
HAC: Humin-derived activated carbon
PXRD: Powder X-ray diffraction
SAED: Selected area electron diffraction
THF: Tetrahydrofuran
TGA: Thermogravimetric analysis
GVL: γ-Valerolactone

## Author contributions

Nivedha Vinod: methodology, validation, analysis, and editing. Saikat Dutta: conceptualization, supervision, and writing original draft.

## Conflicts of interest

The authors declare no competing interest.

## Funding sources

SD thanks DST-SERB, India, for funding under the Core Research Grant (CRG) scheme (File No. CRG/2021/001084 and CRG/2022/009346).

## Supplementary Material
